# Investigating the link between morphological characteristics and diet in an island population of omnivorous reptiles (*Sphenodon punctatus*)

**DOI:** 10.1242/bio.059393

**Published:** 2022-10-14

**Authors:** Sarah K. Lamar, Joseph T. Altobelli, Nicola J. Nelson, Diane K. Ormsby

**Affiliations:** ^1^ School of Biological Sciences, Victoria University of Wellington, Wellington 6012, New Zealand; ^2^ Center for Biodiversity and Restoration Ecology, Victoria University of Wellington, Wellington 6012, New Zealand; ^3^ Zoology Department, University of Otago, Dunedin 9016, New Zealand

**Keywords:** Diet analysis, Feeding ecology, Foraging theory, Reptile diet, Sphenodon punctatus, Stable isotope analysis, Tuatara

## Abstract

The morphological characteristics that impact feeding ecology in ectotherms, particularly reptiles, are poorly understood. We used morphometric measures and stable isotope analysis (carbon-13 and nitrogen-15) to assess the link between diet and functional morphology in an island population of an evolutionarily unique reptile, the tuatara (*Sphenodon punctatus*). First, we established a significant positive correlation between overall body size, gape size, and fat store in tuatara (*n*=56). Next, we describe the relationship between stable isotope profiles created from whole blood and nail trim samples and demonstrate that nail trims offer a low-impact method of creating a long-term dietary profile in ectotherms. We used nitrogen-15 values to assess trophic level in the population and found that tuatara on Takapourewa forage across multiple trophic levels. Finally, we found a significant relationship between gape size and carbon-13 (linear regression: *P*<0.001), with tuatara with large gapes showing dietary profiles that suggest a higher intake of marine (seabird) prey. However, whether body size or gape size is the primary adaptive characteristic allowing for more optimal foraging is yet unknown.

This article has an associated First Person interview with the first author of the paper.

## Teaser

**Summary:** Using a combination of stable isotopes and morphological measures, we assess the feeding ecology of a rare, evolutionarily unique island reptile - the tuatara (*Sphenodon punctatus*).

## INTRODUCTION

Feeding ecology, the theory behind a species’ feeding behaviour and its relationship to the environment, is an important component of community ecology (Gerking, 2014). Many theories seek to explain the patterns observed in feeding ecology across taxa, such as the niche breadth hypothesis and optimal foraging theory (Costa et al., 2008). The niche breadth hypothesis states that species with larger body sizes tend to have more broad geographic distributions (Gaston and Blackburn, 1996) and occupy more diverse microhabitats, therefore consuming a wider variety of prey (Brown, 1984, 1995). In contrast, optimal foraging theory suggests that predators with large body sizes should primarily consume large prey in order to maximize energy intake relative to energy expenditure during hunting (Charnov, 1976; MacArthur and Pianka, 1966). However, these two theories are not mutually exclusive, and may explain patterns of feeding ecology differently across size gradients within a species. For example, different feeding ecologies may be observed across a species’ life history stages or between sexes (Meik et al., 2012; Pearson et al., 2002; Vincent et al., 2004).

Functional traits can also influence a species’ feeding ecology. Many of these traits are reflected by the taxonomic grouping of the study species, such as body size and locomotory method. As a group, the impact of functional morphology on feeding ecology in reptiles is relatively understudied, particularly in island systems. Island ecosystems can be volatile and experience extreme seasonal shifts in occupancy, associated prey, and nutrient availability (MacArthur and Wilson, 1967), and therefore serve as effective mesocosms to observe species at their limits. As island area decreases, large-bodied vertebrates, typically mammalian carnivores, are lost and mesopredators are found in high densities (MacArthur and Wilson, 1967). In the tropics and southern oceans, where native mammals are absent from many islands, these mesopredators are often reptiles (Ortega et al., 2021; Sutherland et al., 2011). Insular reptiles have evolved several adaptations to feeding and digestion relative to their continental conspecifics, such as elongated gastrointestinal tracts, increased gut passage times, increased efficiency of digestion, and gape variation in response to prey composition (Jessop et al., 2006; Sagonas et al., 2015; Vincent et al., 2009). These specialised functional traits have subsequent impacts on a species’ feeding behaviours. Thus, morphological characteristics like body size, gape, and fat store may be helpful to include in diet analyses to better understand the relationship between a species and its prey.

Tuatara (*Sphenodon punctatus*) are long-lived reptiles endemic to New Zealand (Cree, 2014). While superficially similar to lizards, tuatara are the sole surviving members of Rhynchocephalia and possess unique dental characteristics considered diagnostic for the order. First, tuatara have two rows of upper teeth, with one enlarged palatine row running parallel to a row of maxillary teeth; between the two upper rows is a gap that seats the lower teeth when at rest (Evans, 1984; Fraser, 1988). Tuatara also have a bony protrusion on the dentary of the lower jaw and a pair of enlarged teeth at the front of the premaxilla (Fraser, 1988; Reynoso, 2003) (from which the group name Sphenodontia, or ‘wedge tooth’, is derived). While tuatara do have a fleshy tongue, it is connected to their lower palate for the majority of its length (Cree, 2014). Thus, consumption of large prey is determined by the individual's ability to fit its jaws around the prey item, making gape size (derived from jaw width and length) an important differentiating characteristic when analysing feeding behaviour. Smaller prey is consumed in a method unique among living vertebrates, coined the ‘crush and shear’ method, where prey is macerated by characteristic proal jaw action (Cree, 2014). While Rhynchocephalians have occupied as many as five dietary niches across time (Herrera-Flores et al., 2017), tuatara are omnivorous (Cree, 2014; Fraser, 1993; Jones and Cree, 2012).

Extirpated from mainland New Zealand after the arrival of human settlers and the subsequent introduction of invasive mammals, free-roaming populations of tuatara are now restricted to offshore islands (Gaze, 2001). Takapourewa (Stephens Island) is a 1.5 km^2^ (150 ha) predator-free island located in the Cook Strait, New Zealand (Cree, 2014). In addition to hosting the largest remaining population of tuatara (estimated 30,000), Takapourewa is also home to a large abundance of seabirds, invertebrates, and small reptiles (Anderson, 2018; Cree, 2014; Walls, 1978; Tennyson, 1998; East et al., 1995), which all contribute to the omnivorous diet of the tuatara. From approximately July to January, Takapourewa plays host to an estimated 1.83 million pairs of fairy prion (*Pachyptila turtur*), an important source of nutrients for the island's reptiles (Cree, 2014; Walls, 1978). In the winter months, adult fairy prions return to the island and excavate burrows. In spring, eggs are laid. These eggs will hatch in December, with the fledglings leaving Takapourewa by early February (Walls, 1978). Tuatara primarily consume fairy prion eggs and fledgling chicks during the late spring and summer (Walls, 1978); in fact, tuatara are estimated to be the cause of failure of 28% of all fairy prion eggs and chicks on Takapourewa (Walls, 1978). Despite the presence of large seabird colonies, soil on the island varies significantly in the amount of marine-derived carbon and nitrogen present and retains strong isotopic signatures of terrestrial nutrient inputs (Markwell and Daugherty, 2003). While the impacts of marine input throughout the ecosystem may be varied, the contribution of seabirds to tuatara diet is established.

Previous work examining tuatara diet via field observations, scat analysis, and gastric lavage on Takapourewa suggests that tuatara primarily consume herbivorous invertebrates, particularly darkling beetles (*Mimopeus* spp.) and wētā (e.g. *Hemideina crassidens crassicruris, Deinacrida rugosa*) (Fraser, 1993; Walls, 1981). While these are not the only two invertebrate species consumed by tuatara on Takapourewa, they are both relatively large and likely comprise the majority of invertebrate biomass consumed (Walls, 1981). Other common possible prey items found on the island include amphipods (*Orchestia*, *Transorchestia* spp.), various spider species (Order Araneae), Raukawa geckos (*Woodworthia maculata*), and even young tuatara. Additionally, tuatara have been observed eating the fragrant fruit of kawakawa (*Piper excelsum*) (Bredeweg and Nelson, 2010), which are produced in summer. Still, not all tuatara on Takapourewa are thought to consume the same prey. Previous work using stomach flushing and scat analyses suggests that wild post-hatchling and adult tuatara diets differ significantly, but no differences among other life history stages or between size classes of tuatara were detected (Fraser, 1993). However, stomach flushing and scat analyses can be limited in their ability to capture the presence of soft-bodied dietary items, potentially skewing study results. Further, both methods largely represent single time point sampling events. Ectotherms often forage sporadically and many of the food items on Takapourewa are only available seasonally (e.g. cicada eruptions and fairy prion fledging), potentially making such single time point sampling inadequate. Preliminary work using carbon-13 and sample groups of five male, female, and juvenile tuatara on Takapourewa suggested that male tuatara have diets more enriched in marine input (higher carbon-13 signatures) than the other demographic groups (Cree et al., 1999); however, gape size and other morphometric characteristics that may drive variation in dietary consumption over time were not measured and remain unknown for all sex, age, and size classes of tuatara.

Here, we assessed the foraging ecology of an island population of tuatara via stable isotope analysis of carbon-13 (^13^C) and nitrogen-15 (^15^N). First, we assessed how dietary intake varies across timescales by comparing stable isotope profiles generated from two sample types collected from the same individual: nail trim (inert tissue) and whole blood (relatively short protein turnover rate). We hypothesised that the ratio of isotopes would vary between the two sample types due to seasonality in prey availability, particularly seabirds. Next, we analysed the relationship between morphometric characteristics of interest and Δ^13^C, Δ^15^N, and the relative trophic level of each individual using isotope data for potential prey items collected during other studies on Takapourewa or offshore islands in New Zealand. Here, we hypothesised that large tuatara would have higher levels of fat store, larger gapes, and a diet more enriched in predatory taxa, indicative of optimal foraging, relative to smaller tuatara. Finally, we estimated trophic niche width and prey contribution to diet, particularly seabirds, at a population level.

## RESULTS

We captured 41 male and 15 female adult tuatara. Despite displaying sex-based size dimorphism in adults, morphometric values from captured females fell within the range of male measurements, reflecting our intentional sampling along a size gradient. Sampled snout–vent length (SVL) values ranged from 170-277 mm (227.70±4.01) and masses from 170-900 g (490.50±25.95). BCI values ranged from 1.00-1.21 (1.13±0.01). Tail width values ranged from 16-42 mm (29.80±0.80). Jaw width values ranged from 34-61 mm (47.04±0.93), while jaw length measurements ranged from 34-64 mm (49.08±1.01). Finally, GI values fell between 907.92 and 3066.19 (1854.14±71.06).

Linear regressions looking at the relationship between GI and SVL (*P*<0.001; *F*=464.8, d.f.=54), GI and BCI (*P*<0.001; *F*=187.4, d.f.=54), GI and tail width (*P*<0.001; *F*=182.4, d.f.=54), and BCI and SVL (*P*<0.001; *F*=252.2, d.f.=54) confirmed significant, positive relationships among the four variables (Fig. S1).

Considering only raw, uncorrected nail data (*n*=56), Δ^13^C values ranged from −22.62 to –20.66 (−22.28±0.12), while Δ^15^N values ranged from 15.07–20.34 (17.39±0.19). There were no sex-based differences in Δ^13^C (*P*=0.092, t=1.73, d.f.=34.53) or Δ^15^N values (*P*=0.111, t=−1.65, d.f.=27.31). Thus, we did not include sex as a factor in future isotope comparisons. For uncorrected whole blood samples (*n*=10), Δ^13^C values ranged from −25.57 to −22.17 (−24.07±0.30) and Δ^15^N values ranged from 15.17-20.79 (17.13±0.50).

**Fig. 1.**
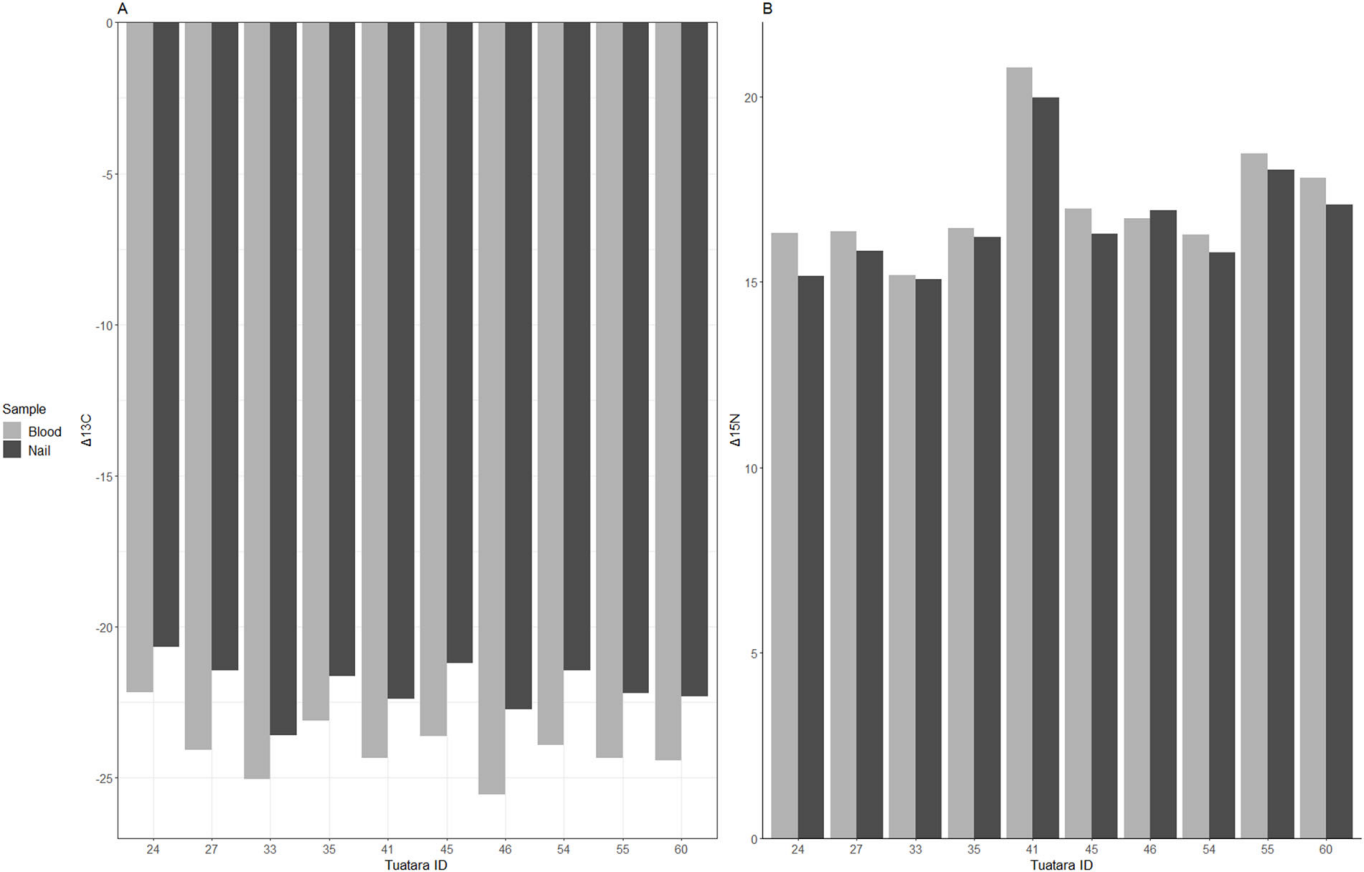
**Raw stable isotope values calculated from different sample types (whole blood versus nail) collected from 10 tuatara (*Sphenodon punctatus*).** A=Δ^13^C and B=Δ^15^N. All stable isotope values shown as expressed as parts per thousand (‰).

There were significant differences between corrected isotope values derived from whole blood and nail samples across individuals (*n=*10): Δ^13^C (*P*<0.001, t=−8.83, d.f.=9) and Δ^15^N (*P*<0.001, t=25.64, d.f.=9). Similarly, there were significant differences in uncorrected isotope data derived from different sample types across individuals (*P*≤0.003, *t*≥−3.98, d.f.=9) (Fig. 1). Residuals for the linear regression comparing isotope values derived from whole blood and nail tissues can be visualised in Fig. S2. The mean difference between uncorrected Δ^13^C values derived from whole blood relative to nail tissue was −2.10 (±0.16). The correlation coefficient for Δ^13^C values derived from different sample types was 0.85. The mean difference between uncorrected Δ^15^N derived from whole blood relative to nail tissue was 0.50 (±0.12). The correlation coefficient for Δ^15^N values derived from different sample types was 0.97.

Relative trophic levels for tuatara ranged from 1.29 to 2.84 (2.02±0.05). The correlation coefficient for GI and Δ^15^N was –0.406.

**Fig. 2.**
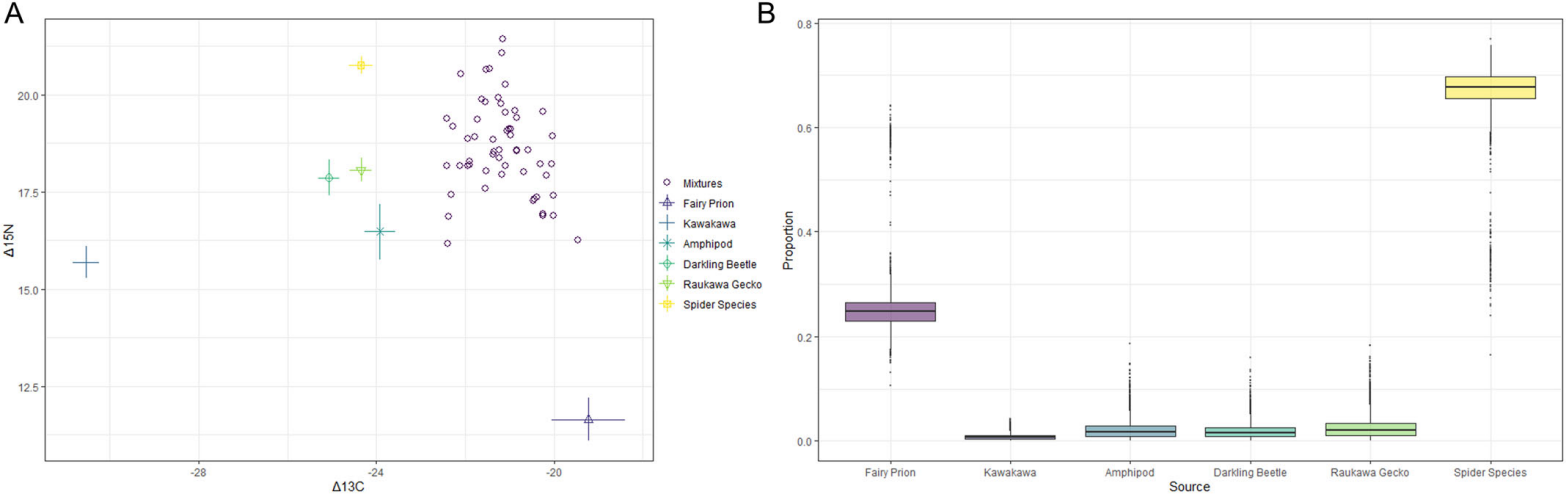
**(A) Isospace plots showing Δ^13^C and Δ^15^N values of 56 tuatara living on Takapourewa, calculated from nail trims (‘mixtures’, hollow points).** Different symbols indicate mean values for prey items, and horizontal and vertical bars indicate standard error for Δ^13^C and Δ^15^N, respectively. Because some sample values fall outside of isospace, this isoplot is not fully resolved. Raw values for Δ^13^C and Δ^15^N have been made available. (B) Approximate contributions of prey to the diet of tuatara living on Takapourewa, calculated as a ‘proportion’ of the whole at the population level from a mixed isotope model (Δ^13^C and Δ^15^N). Calculations assume all prey items contribute to diet and that prey item contributions will sum to one. All isotope values have been corrected to account for tissue-specific fractionation using values from (*Cyclura lewisi*) (further described in-text); plot created using the *simmr* package in R (Parnell, 2021).

Values for Δ^13^C and Δ^15^N calculated from tuatara nail samples fell largely within the isospace (the space defined by isotope values for analysed prey items) (Fig. 2A). However, not all sample values were contained within the convex hull polygon. Importantly, *simmr* proportion models assume all provided prey items contribute to diet and proportions sum to one (Parnell, 2021). Mixing model estimates suggest that spiders compose the majority of tuatara diet on Takapourewa (65.3%), with fairy prions being the next largest contributor (26.9%) (Fig. 2B). Amphipods, darkling beetles, and Raukawa geckos are estimated to each compose between 2.0-2.7% of the diet of tuatara on Takapourewa, with kawakawa composing only <1% of total dietary intake (Fig. 2B). While not discussed, the location of blood-derived stable isotope samples is visualised in isospace in Fig. S3.

Using the average raw whole blood Δ^13^C value (–24) to calculate the contribution of fairy prion to tuatara diet yielded a result of 19.4%. Using the corrected value for whole blood Δ^13^C (−22.16), we calculated that fairy prion likely comprise 40.0% of tuatara diet on Takapourewa in our sampling period.

**Fig. 3.**
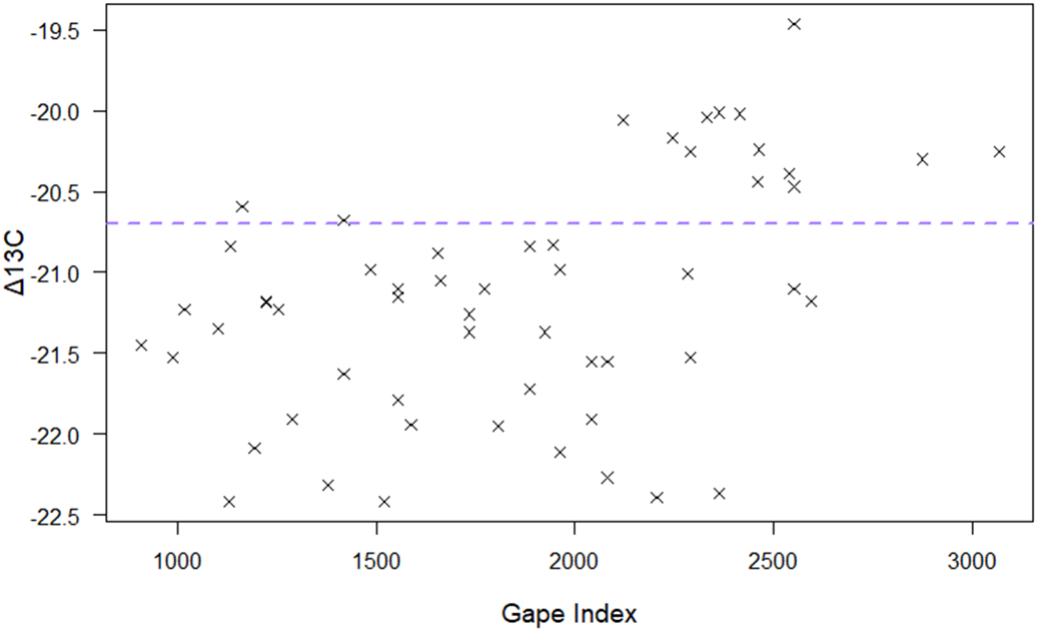
**Tuatara Δ^13^C values plotted against GI (*n*=56); the horizontal line demarks the Δ^13^C values for fairy prion on Takapourewa (−20.7) (Cree et al., 1999).** Tuatara with a Δ^13^C value higher than fairy prion, indicating a large marine influence on diet, had GI that were significantly different than those with lower Δ^13^C values (Welch's *t-*test, *P*=0.0002, *t*=4.50, d.f.=23.08).

Finally, a linear regression indicated a significant positive relationship between gape size and Δ^13^C (*P*<0.001, *F*=14.04, d.f.=54, slope=6.250e-04). Furthermore, tuatara with a Δ^13^C value higher than fairy prion, indicating a large marine influence on diet, had gape indices that were significantly larger than those with lower Δ^13^C values (*P*<0.001, *t*=4.50, d.f.=23.08) (Fig. 3).

## DISCUSSION

Our research investigating the relationship between morphology and prey consumption in an insular population of an omnivorous reptile, the tuatara, has offered insight into the evolution of reptile foraging systems. We confirmed a strong positive relationship between snout–vent length, body condition, tail width, and gape index, indicating that tuatara with longer snout–vent lengths have larger gapes, are in better body condition, and have larger fat stores than shorter tuatara. The reasons for this are likely twofold and interactive. First, tuatara on Takapourewa with larger gapes have access to higher quality prey items, like the seabirds that seasonally inhabit the island. Seabirds, including the fairy prion, are enriched in C20 and C33 polyunsaturated fatty acids, particularly eicosapentaenoic and docosahexaenoic acids (Cartland-Shaw et al., 1998). These polyunsaturated fatty acids are important for egg hatchability, embryo development, and juvenile growth in reptiles (Cartland-Shaw et al., 1998; Lawniczak and Teece, 2009; Noble et al., 1993; Speake et al., 1994; Staton et al., 1990), and in males of other taxa are involved in oocyte signalling controlling sperm motility direction (Kubagawa et al., 2006) and sperm storage (Lahnsteiner et al., 2009). While the polyunsaturated fatty acid content of other possible prey species on Takapourewa, such as spiders, is not known, polyunsaturated fatty acids level trend higher in aquatic systems, both freshwater and marine, than terrestrial systems (Chari et al., 2020; Colombo et al., 2017; Mathieu-Resuge et al., 2022); for example, emergent freshwater aquatic invertebrates have levels of eicosapentaenoic acid as much as 6.6 times enriched relative to terrestrial insects (Mathieu-Resuge et al., 2022). Further, high latitude marine organisms provide long-chain polyunsaturated fatty acids at rates disproportionately high relative to terrestrial consumers (Colombo et al., 2017). Additionally, the levels of polyunsaturated fatty acids in spiders have been found to mirror their diet, though species-specific analyses are needed to confirm this for spiders on Takapourewa. Regardless, the adapted hindguts often found in populations of insular reptiles maximise prey energy extraction, and likely compound the effects of the consumption of prey rich in polyunsaturated fatty acids and other valuable nutrients to a greater extent than in their continental conspecifics (Jessop et al., 2006; Sagonas et al., 2015; Vincent et al., 2009).

The second possible explanation for the links between morphometric characteristics in tuatara is that large tuatara may have access to a greater quantity of prey. This could be because a larger gape allows for the ingestion of a broader size range of prey items, or because large male tuatara consistently win aggressive interactions against smaller tuatara (Moore et al., 2009) and thus may be better competitors for food. In fact, as body size (SVL) increases, so does the likelihood that a male tuatara living on Takapourewa will be able to hold an exclusive territory in the densely populated forest area (Moore et al., 2009). The selection for increased gape size to allow for access to a wider variety of prey items may drive a non-adaptive increase in body size and an understanding of tuatara dietary composition is needed to clarify which of these variables, or an interactive effect, is driving increased fat store. These explanations for the correlation between body length, body condition, tail width (as a proxy for fat store), and gape size are not mutually exclusive.

Next, we analysed Δ^13^C and Δ^15^N values calculated from different sample types (nail trim versus whole blood). We found that isotope values varied significantly between sample types, likely reflecting different protein turnover rates. Whole blood protein turnover rates in ectotherms are slower than those of endotherms and vary widely (e.g. 2-6 months) (Hobson and Clark, 1993; Seminoff et al., 2007). However, these samples still offer a shorter-term snapshot of dietary intake relative to other tissues and are useful for investigating diet at specific time points. In contrast, nail tissue represents a longer-term estimate for dietary intake. While nail growth is a linear process (Orentreich et al., 1979), nail tissue is inert and the stable isotope values embedded in the nail tissue as it leaves the nail bed are preserved (Bearhop et al., 2003). Thus, nail trims of different lengths can represent different time periods of dietary intake and be used for serial sampling (Hénaux et al., 2011). However, as the growth rate of tuatara nail is unknown and likely impacted by wear and individual behaviour, such as female nest digging, we do not estimate the timescale at which our dietary analyses occur. Despite this, correlation coefficients for both uncorrected isotope values were significant (α>0.80), suggesting that the turnover time for stable isotope incorporating proteins in tuatara whole blood may be protracted and present a dietary snapshot over a moderate timescale. Further, this relationship confirms that dietary trends observed in nail samples are accurate to the individual, even when different time scales are considered. In summary, we have found that nail trims are an easy, low impact method of stable isotope sampling for tuatara, and have described the suspected conversion factor for Δ^13^C and Δ^15^N derived from tuatara whole blood and nail tissue.

To calculate the relative trophic level of tuatara living on Takapourewa, we compared Δ^15^N values from tuatara samples to plant values at the base of the island's terrestrial food web (*P. excelsum, C. repens,* and *L. perenne*). Our results suggest that tuatara forage between 1.29 and 2.84 trophic levels above base, indicating a sizable range of prey consumption within the population. Unexpectedly, trophic level and gape index were negatively correlated, albeit not significantly. However, the difference in Δ^15^N_base_ values between terrestrial and marine systems makes interpretation of the direction of these trophic level trends difficult (Post, 2002). Further, calculating trophic level from Δ^13^C values alone is difficult, as rates change at an estimated >1% per level (Post, 2002). Therefore, we issue a note of caution at interpreting the herein calculated trophic levels, but nevertheless present an idea of the variation in trophic level being consumed between individuals in this population.

While many mixed isotope samples fell within the convex hull polygon of our *simmr* models (Fig. 2A), many did not. We offer the three most likely reasons for why this may be: (1) stable isotope reference values for possible prey items were not accurate, (2) possible prey items are missing from our model, or (3) our tissue-specific fractionation value, taken from the scute of a caiman, is a poor surrogate for this species. For example, spiders (order Araneae) were the only terrestrial carnivore in our models. However, on Takapourewa, there are also four skink species which would fall into the category of terrestrial predators: speckled skink (*Oligosoma infrapunctatum*), northern spotted skink (*O. kokowai*), northern grass skink (*O. polychroma*), and the glossy brown skink (*O. zelandicum*). Tuatara on Takapourewa have been observed eating skinks (Fraser, 1993), which can grow as large as 106 mm (SVL; *O. infrapunctatum*; van Winkel et al., 2018). All skink species on Takapourewa are primarily insectivorous, but will occasionally consume soft fruits and small lizards, including geckos (van Winkel et al., 2018). Further work including these skink species may help resolve the model. Regardless, we feel that the results of this *simmr* model are a valuable addition to the field, and challenge the previous theories that tuatara on Takapourewa are foraging primarily on soft-bodied herbivorous insects. However, more work to address the concerns raised above, and resolve the remaining issues with isospace alignment, is needed. Thus, we present the isoplot data (Fig. 2), but do not further expand on the interpretation of those results.

Our dietary proportion estimates suggest the population average for contribution of fairy prions to tuatara diet is 26.9%. Previous work using whole blood Δ^13^C found that 45±16% of sample carbon collected from tuatara in February was from fairy prions (Cree et al., 1999); our results suggest a similar value of approximately 40%. Three non-exclusive factors likely influence the ability to compare these results. First, the previous study was conducted prior to any fractionation values for reptiles being available, highlighting a taxonomic bias in historical isotope data availability. Instead, it used values from laboratory rats (*Rattus norvegicus domestica*) and American crows (*Corvus brachyrhynchos*). The ^13^C fractionation value calculated for blue iguana blood (1.7) used in this study differs significantly from the fractionation factor previously used to calculate ^13^C in tuatara blood samples (0.4) (Cree et al., 1999). Second, the previous study used only five adult males, whereas our study included the whole blood of 10 adults. Finally, the previous study was conducted at the peak of fairy prion fledging (February) using whole blood samples, which have short protein turnover rates. During our study, no fairy prion fledgelings were seen on the island, and thus, due to the turnover rate of whole blood, may represent only the signature of fairy prion consumption post-fledgeling season.

Finally, linear regression indicated a significant relationship between gape size and Δ^13^C, with an increase in gape size resulting in an increase in Δ^13^C (less negative values). Thus, at an individual level, tuatara with larger gapes have a Δ^13^C signature that suggests a diet more rich in marine (seabird) input, which was confirmed with a *t*-test. The ability of larger tuatara to access nutrient-rich seabirds as a food source may be why tuatara body condition and tail width increased with SVL, indicating an increase in fat store relative to smaller tuatara. However, previous work exploring the relationship between diet, gape, and body size in insular populations of a snake (*Elaphe quadrivirgata*) suggested that body size did not coevolve with diet, but rather that variation in body size among populations of *E. quadrivirgata* was in part due to underlying phenotypic covariation with gape size, which closely matched the size of major dietary items in each population (Vincent et al., 2009). Importantly, we suggest that future work examines variation in gape, not just body length, among tuatara populations, as body length (and overall size) may be a spandrel of the evolution of gape size to match prey composition. Further, research investigating the feeding ecology of the mangrove water snake (*Nerodia fasciata compressicauda*) found that large snakes were more selective about prey size than small snakes, despite being able to consume a wider size range of prey items (Miller and Mushinsky, 1990). Rather counterintuitively, large snakes consumed smaller prey relative to their calculated gape index, likely in an effort to reduce the time spent foraging, and therefore time vulnerable to predators (Miller and Mushinsky, 1990). Research considering time spent foraging, in addition to the nutrient benefits of prey discrimination, should be undertaken. Further, male tuatara are significantly larger than females, defend their territories aggressively, and engage in long courting displays (known as the ‘stolzer Gang’, a term coined by Gans et al., 1984). This, combined with research suggesting that spermiogenesis is a more energy intensive process than previously thought (e.g. Olsson et al., 1997), may mean that male tuatara on Takapourewa have higher energy requirements than the females living in their territories, especially when sampled during the peak of their spermiogenesis, territoriality, and courting behaviours (mating season, February to March) (Cree et al., 1992; Saint Girons and Newman, 1987; Moore et al., 2009). However, we cannot rule out that only male tuatara forage optimally, with female tuatara foraging opportunistically, leading to differences in isotope signature along a size gradient. The sexually size-dimorphic nature of tuatara make this a difficult distinction to parse, particularly in an *in situ* study. Nevertheless, we offer it as an alternative explanation that cannot be excluded. Regardless, these results provide empirical support for the development of the theoretical principle that male tuatara forage in a manner consistent with optimal foraging theory.

Tuatara size varies greatly among different populations, with larger animals consistently being found on more northern, warmer islands like Tawhiti Rahi (35.456° S) (Cree, 2014). For example, during one sampling year males on Tawhiti Rahi reached SVLs of 311 mm and weighed as much as 1.1 kgs, while in the southernmost relict population of tuatara on North Brother Island (41.114°S), males reached SVLs of only 256 mm and weighed at most 655 g during the same year (Cree, 2014). Importantly, recent genome sequencing has shown that the tuatara of North Brother Island, once their own species but now synonymized with the senior synonym, are genetically distinct (Gemmell et al., 2020). Possessing 8480 unique alleles, the conservation of this struggling population remains a priority (Gemmell et al., 2020; Lamar et al., 2021). Despite being only four hectares, North Brother Island plays host to many seabirds, chiefly the common diving petrel (*Pelecanoides urinatrix*). Diving petrels are smaller than fairy prion (20 cm versus 25 cm length) (Miskelly, 2013a,b), possibly making them more accessible to the small tuatara that live on the island. Interestingly, the body sizes of insular populations of snakes are often bimodal, with gigantism occurring on islands with large prey sizes and dwarves on islands with smaller prey (Boback, 2003; Vincent et al., 2009). It is unknown if this is occurring to some degree across populations of tuatara, and further work is needed to examine what seabirds are consumed by tuatara at their range extremes and the fitness implications of these varied nutrient sources on different size classes, and sexes, of tuatara. Interestingly, tuatara have been restored to several predator-free, mainland sanctuaries. While research shows that seabird-inhabited islands consistently have greater prey abundance than those without (Markwell and Daugherty, 2003), the lack of seabird colonies and associated nutrient sources on tuatara fitness has yet to be examined for these populations.

Larger tuatara have diets comparatively rich in seabirds, most likely a result of their increased gape size and ability to outcompete smaller individuals for quality prey items. However, whether body size or gape size is the primary adaptive characteristic is yet unknown. More research investigating the feeding ecology of tuatara in different populations across their range is needed to further clarify the breadth of variation in body size and diet across size classes for this unique species.

This work informs our understanding of reptile foraging using an evolutionarily unique reptile endemic to an island ecosystem that evolved in the absence of any mammalian mesopredators. Importantly, we emphasise the need for research on reptile mesopredators to consider gape size and a measure of fat store when conducting research on foraging ecology. Much of the current reptile literature does not consider that body size may be serving as a spandrel of evolving gape, particularly in island populations, and whether this selection pressure may differ between size dimorphic sexes as a function of sex-based differences in behaviour or simply size class. To our knowledge, no work looking at this distinction in non-ophidian reptiles has been conducted. Nevertheless, this work offers new insights into the diet and morphometric characteristics of reptiles that persist in extreme, high-pressure environments and is important for our understanding of the functional morphology of reptilian feeding ecology.

## MATERIALS AND METHODS

### Sample collection

Research was carried out under Wildlife Act Authority permit #50568-FAU and Victoria University of Wellington animal ethics committee permit #30011.

In February-March of 2021, we undertook nightly visual surveys for tuatara on Takapourewa. We focused sampling in the central, forested area of the island known as ‘Keeper's Bush’, where adult tuatara can be found in densities as high as 2732 tuatara per hectare (Moore et al., 2009). Juveniles (approximately <15 years of age) are not commonly encountered in this area of high competition, so we selected adult tuatara that represented a range of size classes. From each individual (total *n*=56), we collected a suite of morphometric data, including: SVL, tail width at the widest point (as a proxy for fat store, Cree, 2014), jaw width, jaw length, and mass. We also collected 1-2 nail trims (≥0.001 grams) from the distal end of the longest nails on the rear feet using a standard pet nail trimmer. Finally, we drew a whole blood sample (approximately 0.4 ml) from the ventral coccygeal vein of a subset of individuals being included in another study (all male, *n=*10); after drawing, a layer of 100% ethanol was added to each blood vial. We stored both nail trims and whole blood samples at room temperature and released individuals at their capture location.

Prior to sample analysis, we sonicated nail trims in a 1:1 dichloromethane:methanol solution to remove debris. Samples were analysed for Δ^13^C and Δ^15^N at the Institute of Geological and Nuclear Sciences (GNS Sciences), Te Pū Ao (Lower Hutt, New Zealand). There, blood samples were dehydrated in a 40°C drying oven. All samples were weighed in tin capsules and analysed via combustion using a Eurovector (Pavia, Italy) elemental analyser coupled to an Isoprime (IL, USA) mass spectrometer. Isotope values provided are enrichment or depletion relative to a standard; the analytical precision for reported values is 0.3% for Δ^15^N and 0.2% for Δ^13^C. The units for isotope values are parts per thousand (‰).

Stable isotope data (Δ^13^C and Δ^15^N; mean±s.e.) for possible prey items (e.g. vegetation and invertebrates), excluding fairy prion, were taken from a previous study which examined terrestrial trophic webs on Takapourewa (Markwell, 1999). Stable isotope data for fairy prion were taken from studies conducted on predator-free Motunau Island (Hawke and Clark, 2010; Hawke et al., 2005). While Motunau Island is not in the Cook Strait, the average fairy prion Δ^13^C value for that study (mean±standard deviation: −19.23±0.83 ‰), calculated from feather, bone, and collagen, aligned closely with the only isotope data available for fairy prion on Takapourewa (−20.7±0.4 ‰) (Cree et al., 1999), for which there is no existing Δ^15^N value. No Δ^15^N data were available for tree wētā.

### Statistical analyses

To standardise for the effects of tail loss and sex, we calculated body condition index (BCI) (Hoare et al., 2006):
(1)

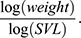
Similarly, we calculated gape index (GI) as follows (King, 2002):
(2)

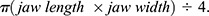
We conducted all statistical analyses using R version 4.1.0 (R Core Team, 2021). We report summary values as means±standard error (s.e.), calculated using the *psych* package in R (Revelle, 2021). We set the alpha level for significance testing at α=0.05.

As there are no tissue fractionation values available for any isotope collected from tuatara, we opted to use the mean blood fractionation values calculated for juvenile blue iguanas (*Cyclura lewisi*) (*n=*15, mean±SD SVL (mm)=187.3±51.8 and mass (kg)=0.3±0.4): Δ^13^C=1.7 and Δ^15^N=2.9 (Steinitz et al., 2015). Broad-scale assessments of stable isotope turnover rates found that the strongest predictors of tissue-specific fractionation values were animal group and body mass (Vander Zanden et al., 2015); blue iguanas are the closest in size to tuatara of the reptile species for which different tissue fractionation values are known, which are mostly turtle species and crocodilians (e.g. Steinitz et al., 2015; Marques et al., 2014), making blue iguanas a good candidate for fractionation estimation in this species. Nail (claw) fractionation values are much scarcer in the literature but are expected to vary significantly from dermal (skin/scute) samples due to differences in tissue turnover rates; unfortunately, there are no existing formulas for calculating claw tissue fractionation values using body mass or taxa. For this reason, we did not use skin fractionation values from *C. lewisi*, but instead used the only claw fractionation values readily available for a reptile – the broad-snouted caiman (*Caiman latirostris*): Δ^13^C=1.2 and Δ^15^N=1.1 (Marques et al., 2014).

**Fig. 4.**
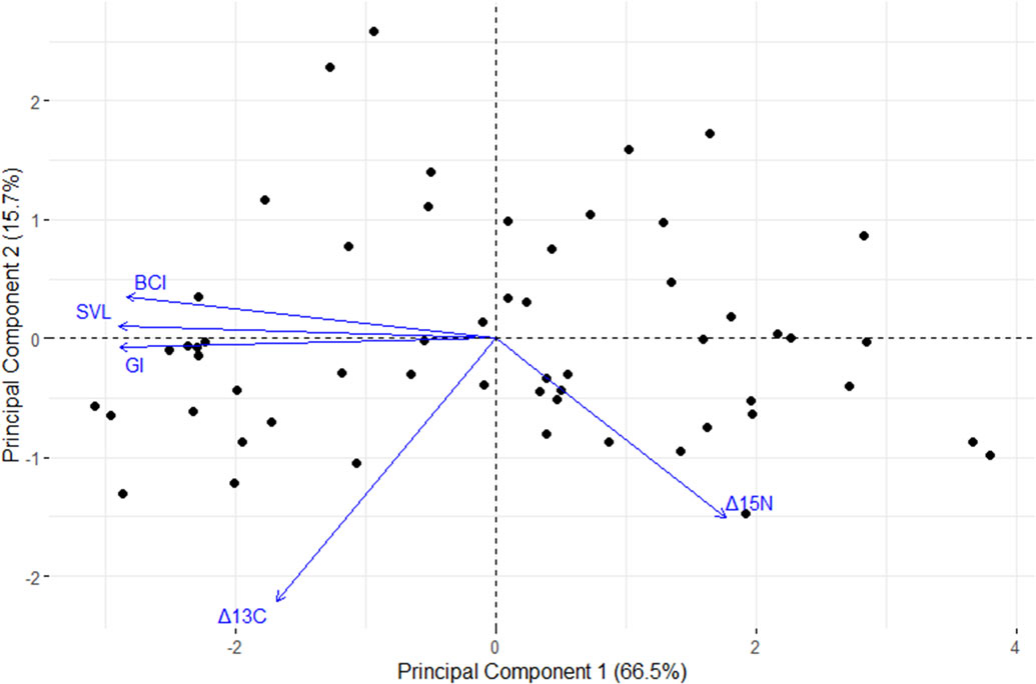
**Principal component analysis (PCA) of three primary morphometric characteristics for tuatara.** (*n*=56), BCI (see Eqn 1), SVL, and GI (see Eqn 2) and stable isotope ratios for Δ^13^C and Δ^15^N. Plot was created using the *factoextra* package (Kassambara and Mundt, 2020) in R.

To test the relationships between SVL, BCI, tail width, and GI, we ran a series of linear regressions. Next, we visualized the relationship between SVL, BCI, and GI with Δ^13^C and Δ^15^N values using a PCA created using the *factoextra* package (Kassambara and Mundt, 2020) (Fig. 4).

We tested for sex-based differences in isotope values (using nail data) with Welch's *t* tests. We compared Δ^13^C and Δ^15^N mean values from whole blood and nail samples collected from the same individuals (*n*=10) using paired *t*-tests for both corrected and uncorrected isotope values. To investigate trends in the relationship between whole blood and nail isotope values for each individual, we ran linear regressions between sample types for each isotope, visualised the linear regression residuals, and calculated correlation coefficients.

To calculate relative trophic level, we used the following formula (Hobson et al., 1994):
(3)

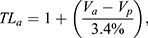
where TL_a_=trophic level of focal animal, V_a_=Δ^15^N of the focal animal, V_p_=Δ^15^N of study site plant tissue, and 3.4% is the estimated increase in Δ^15^N with each trophic level associated with trophic fractionation (Cabana and Rasmussen, 1994). We used historic average data from samples of three plant species (*P. excelsum*, *Coprosma repens*, and *Lolium perenne*) collected on Takapourewa for V_p_ (mean Δ^15^N=15.18) (Markwell, 1999). We then calculated the correlation coefficient for GI and Δ^15^N.

To visualise population trophic niche width, we used the R package *simmr* (Parnell, 2021) to create isoplots of: tuatara nail samples, kawakawa (Markwell, 1999), amphipod (Markwell, 1999), darkling beetle (Markwell, 1999), Raukawa gecko (Markwell, 1999), spider (Markwell, 1999), and fairy prion (Hawke and Clark, 2010; Hawke et al., 2005) isotope data (mean±s.e.), with Δ^13^C as abscissa and Δ^15^N as the ordinate. We assessed prey contribution to tuatara diet, as a proportion, by calculation of the probability that the contribution to dietary proportion of one source is larger than the other (Parnell, 2021).

We compared the contribution of seabird carbon in our whole blood Δ^13^C values against the only other whole blood Δ^13^C calculated for tuatara using the following equation (Cree et al., 1999):
(4)

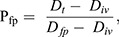
where P_fp=_the proportion of fairy prion in the sample, D_t_=Δ^13^C for the tuatara sample, D_iv_=Δ^13^C of a tuatara consuming solely herbivorous invertebrates, and D_fp=_Δ^13^C of a tuatara consuming solely fairy prion.

Finally, we explored the relationship between gape size and fairy prion intake by visualising the relationship between Δ^13^C and GI. We grouped individuals with a higher (less negative) Δ^13^C than the island-specific fairy prion value (−20.7) (Cree et al., 1999) and compared their GI against tuatara with a lower (more negative) Δ^13^C value than fairy prion via a Welch's two sample *t*-test for unequal variance and ran a linear regression.

## Supplementary Material

10.1242/biolopen.059393_sup1 Supplementary information
