# Supplementary material for: Investigating the link between morphological characteristics and diet in an island population of omnivorous reptiles (Sphenodon punctatus)
Source: Biol Open. 2022 Oct 14;11(10):bio059393. doi: 10.1242/bio.059393 (PMC9581517; doi:10.1242/bio.059393)
Supplement: Supplementary information [file biolopen-11-059393-s1.pdf]

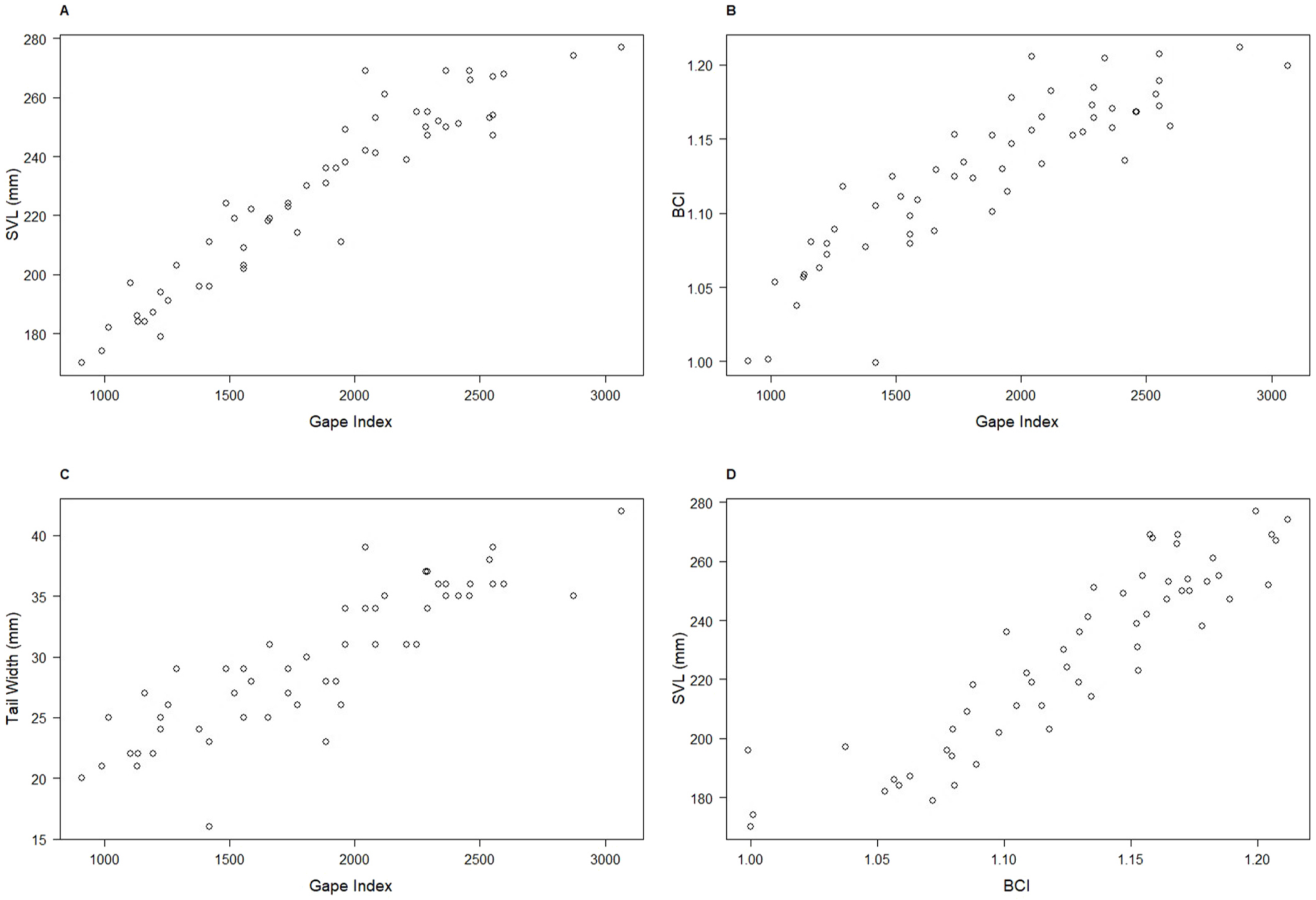

Fig. S1. Plots visualizing linear regressions between key morphometric measures (*n*=56): (A) gape index (GI) and snout-vent length (SVL) ($p < 0.001$; $t = 21.56$), (B) GI and body condition index (BCI) ($p < 0.001$; $t = 13.69$), (C) GI and tail width ($p < 0.001$; $t = 13.51$), and (D) BCI and SVL ($p < 2e-16$; $t = 15.88$).

Biology Open • Supplementary information

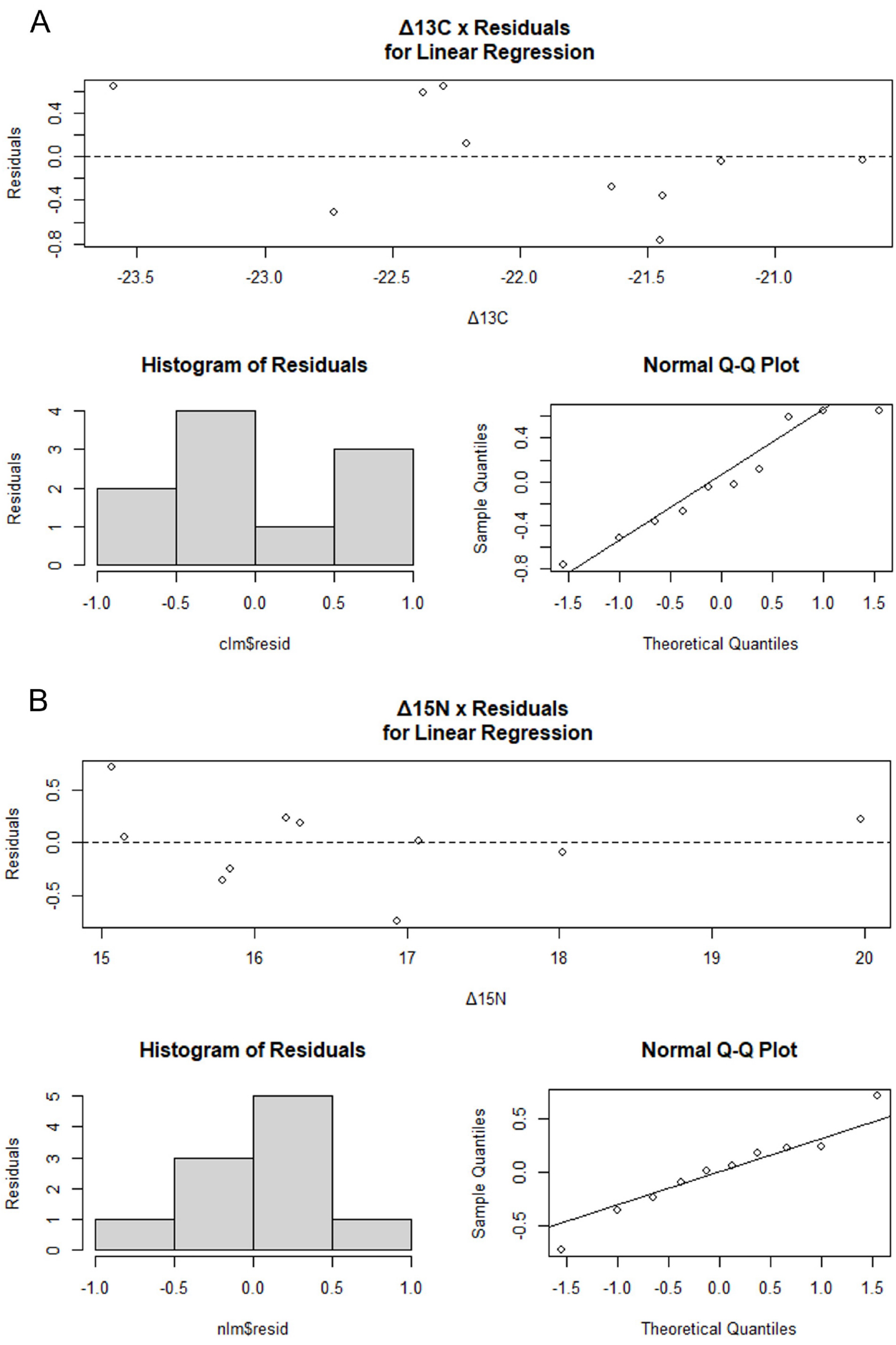

Fig. S2. Panel plots displaying the relationship between residuals for raw, uncorrected nail and whole blood isotope values collected from the same individual (*n* = 10). (A) The correlation coefficient for $\Delta^{13}C$ sample types was 0.85. Where y = blood value and x = nail value, the model formula for $\Delta^{13}C$ values derived from different tissue types can be expressed as $y = -3.0165 + 0.9585(x)$. (B) The correlation coefficient for $\Delta^{15}N$ calculated from different sample types was 0.97 and the model formula for $\Delta^{15}N$ derived from different tissue types is $y = -0.4408 + 1.02716(x)$.

Biology Open • Supplementary information

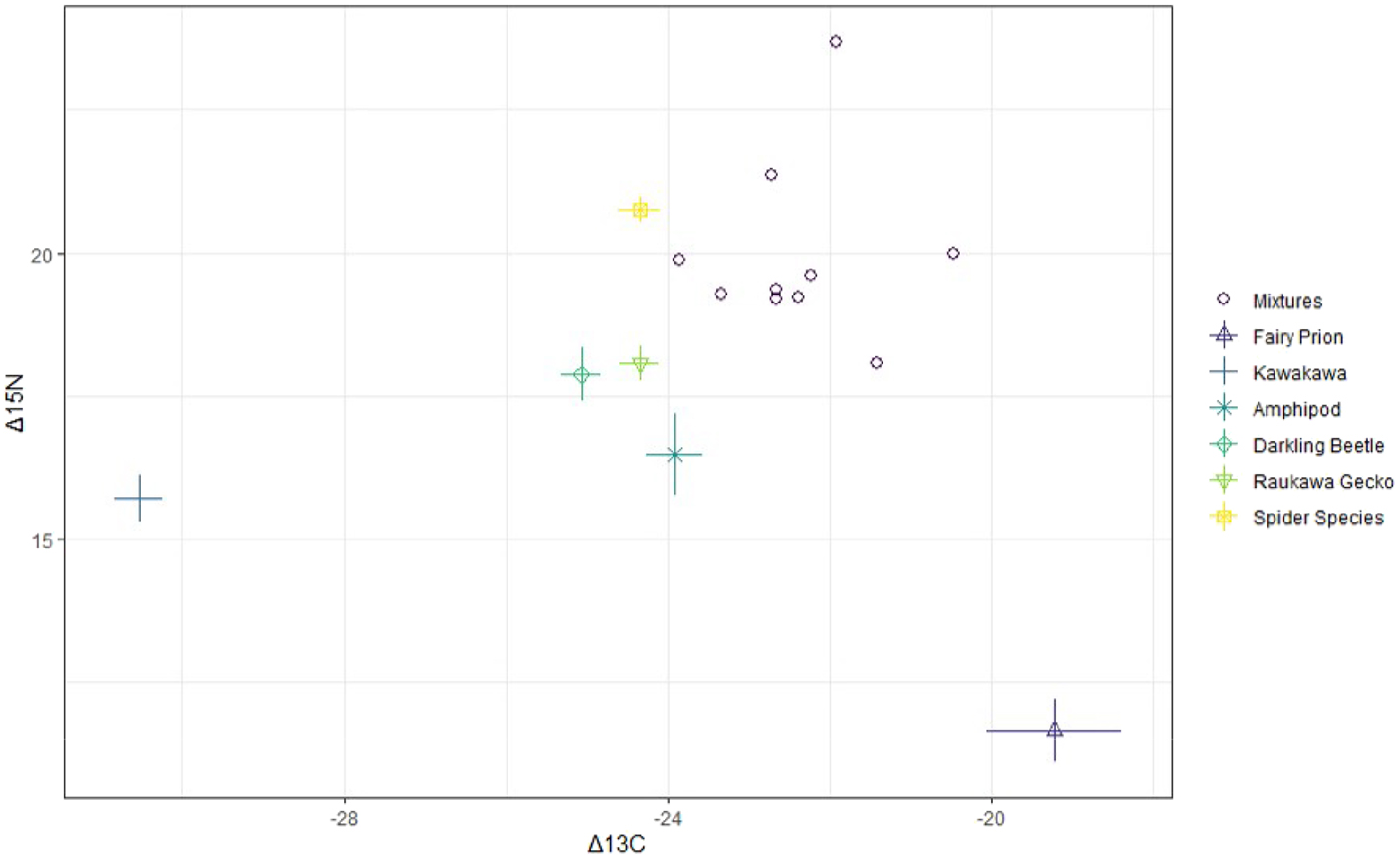

Fig. S3. ***Simmr*** **model results visualizing tuatara blood-derived stable isotope values, and possible prey items, calculated in this study (*n*=10).**

Biology Open • Supplementary information
